# CHANGES IN SOCIAL CONTACTS AND PSYCHOLOGICAL WELL-BEING AMONG OLDER ADULTS DURING THE PANDEMIC

**DOI:** 10.1093/geroni/igae098.2327

**Published:** 2024-12-31

**Authors:** BoRin Kim, Ke Li, Chung Hyeon Jeong

**Affiliations:** University of New Hampshire, Durham, New Hampshire, United States; University of New Hampshire, Durham, New Hampshire, United States; University of New Hampshire, Durham, New Hampshire, United States

## Abstract

Social interactions are critical for health and well-being among older adults. The pandemic has significantly reduced face-to-face social contacts while promoting remote interactions. This study explored the patterns of changes in in-person and remote social contacts during the first year of the pandemic, and their impacts on psychological well-being among older adults. Also, we examined if remote social contacts could substitute for in-person contacts in terms of psychological well-being. Data came from older respondents (age65+) in the 2021 Health and Retirement Study Perspectives on the Pandemic, focusing on the first-year pandemic experiences (N=4,539). Latent class analysis identified distinct patterns of changes in in-person and remote contacts with diverse social relationships. OLS regressions examined the associations between changes in social contacts and psychological well-being. Four profiles were derived for changes in in-person and remote contacts, respectively. While “Decreased All-Types Contacts” was the largest among in-person contact profiles (36.8%), most of our sample was categorized into “Stable Contacts” among remote contact profiles (64.0%). Decreasing in-person contacts were negatively associated with well-being regardless of the types of relationships. Interestingly, older adults experiencing increased remote contact with friends and/or diverse groups were likely to have worse psychological well-being. Interaction analysis showed that remote social contacts served as an alternative to in-person contacts only for the relationship with friends. Our findings highlight the challenges that older adults faced in maintaining social connections through non-traditional channels during the pandemic and underscore the need for targeted support for older adults in the dynamic changes of social landscapes.

